# Dermoscopic findings in human monkeypox infection^☆^

**DOI:** 10.1016/j.abd.2022.10.002

**Published:** 2022-11-18

**Authors:** Leandro Ourives Neves, Amanda Domingos Cordeiro, Bruna Dell’Acqua Cassão Rezende

**Affiliations:** Hospital das Clínicas, Universidade Federal de Goiás, Goiânia, GO, Brazil

Dear Editor,

Currently, Brazil and several other countries are reporting a rapid increase in *Monkeypox* Infection (MPX) cases, especially in Men who have Sex with Men (MSM), with no apparent epidemiological links to endemic areas, representing an important global public health concern.^1^ This emerging zoonotic disease, previously seen only in West and Central Africa, is an *Orthopox* virus transmitted through intimate contact and air droplets, with the possibility of spreading via sexual fluids.^2^, ^3^ A 37-year-old patient, identifying himself as an MSM, presented to the urgency unit at our University Hospital, for a light pruritic cutaneous eruption that had appeared 4 days before, with multiple vesicular papules or erythematous-exulcerated pustules on limbs, face, trunk, penis, and perianal region, some with marked umbilication and central crusting (Figure 1, Figure 2). He also presented with left cervical lymphadenopathy. Prior to the appearance of skin lesions, he had reported headache, low-grade fever, and malaise for 2 days. The patient did not recall being in close contact with animals and denied traveling abroad but mentioned some occasions of condomless sexual intercourse in the preceding weeks. Dermoscopy showed whitish structureless areas with brownish central crusts or ulcerations and perilesional erythema (Figure 3, Figure 4).^4^ After the clinical examination, notification of a suspected case of MPX was performed, blood tests were taken and the collection of scrapings or fluid from the floor of the lesions to detect *Monkeypox* (MP) DNA using the Real-Time Polymerase Chain Reaction (RT-PCR) assay was scheduled. The patient was discharged home, with all contact and droplet isolation measures guidelines. Serology examination was positive for syphilis (VDRL 1:1024), and also reactive for HIV (rapid test and immunoassay); tests for hepatitis B and C were non-reactive; RT-PCR assay for MP was positive.

**Figure 1:**
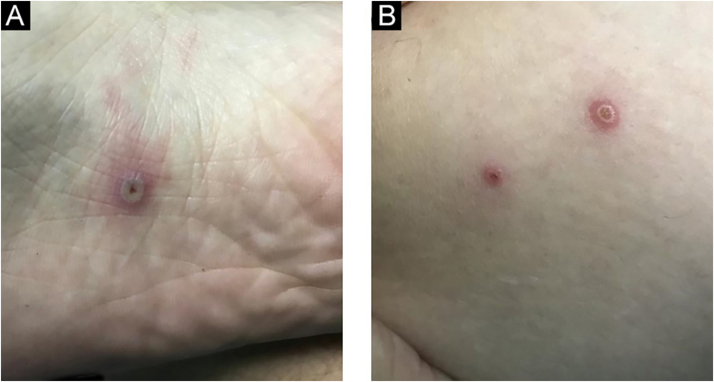
Vesiculopustular lesions with central ulceration at the plantar surface (A), and thigh (B).

**Figure 2:**
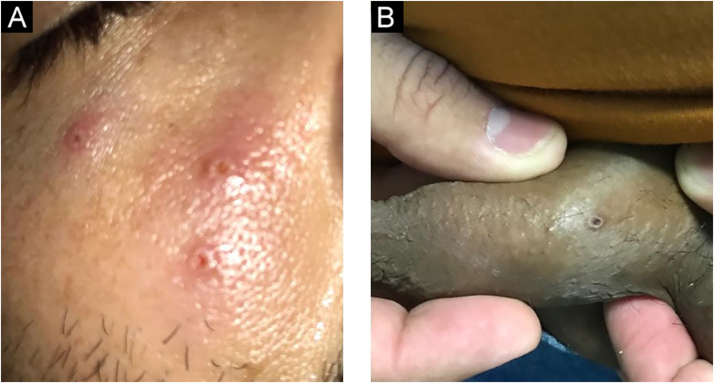
Typical MPX vesiculopustular lesions with central ulceration at face (A) and penis (B).

**Figure 3:**
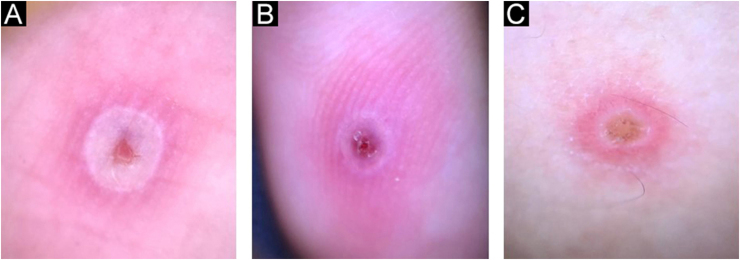
Dermoscopic images of MKX showing ulcerated pink or crusted brownish central area, with white peripheral halo and perilesional erythema at the plantar surface (A), index finger (B), and thigh (C).

**Figure 4:**
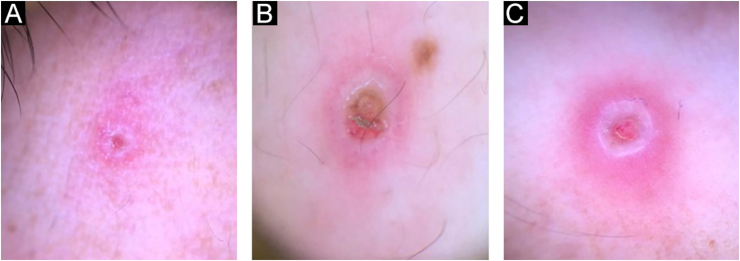
Dermatoscopic features of MKX lesions: central structureless pinkish or with brown crusting area, with white peripheral halo, circulated by pink clods and perilesional erythema, at face (A), penis (B), and perianal area (C).

Some of the symptoms of this patient, prior to the initiation of antibiotic therapy, could also be considered concurrent manifestations of syphilis.^5^ The patient received a prescription for treatment of secondary syphilis with benzathine penicillin and was referred to an infectious disease specialist to start treatment for HIV.

Therefore, the currently dominant interhuman spread in MSM with possible other Sexually Transmitted Infections (STI) coinfections is a valid cause for better awareness of MPX in dermatovenerologic settings, as the patient might seek those prior to visiting other specialists. Recently MPX is spreading rapidly in the world, especially due to MSM. These patients often have combinations of several STIs. So, it is necessary to consider the diagnosis of MPX in all MSM patients with typical rash and risky sexual behavior. For these cases, it is important to ensure accessible, rapid, and reliable tests to prevent the further spread of the diseases. Dermoscopy could be a very useful supplementary diagnostic method in the evaluation of MPX and other viral skin infections.^6^, ^7^

## Financial support

This research did not receive any specific grant from funding agencies in the public, commercial, or not-for-profit sectors.

## Authors’ contributions

Leandro Ourives Neves: Article design; article organization; drafting of the manuscript; review and approval of the final version of the manuscript.

Amanda Domingos Cordeiro: Drafting and editing of the manuscript; review and approval of the final version of the manuscript.

Bruna Dell’Acqua Cassão Rezende: Drafting and editing of the manuscript; review and approval of the final version of the manuscript.

## Conflicts of interest

None declared.
